# Supplementary material for: A Critical View of the Application of the APEX Software (Aqueous Photochemistry of Environmentally-Occurring Xenobiotics) to Predict Photoreaction Kinetics in Surface Freshwaters
Source: Molecules. 2019 Dec 18;25(1):9. doi: 10.3390/molecules25010009 (PMC7017383; doi:10.3390/molecules25010009)
Supplement: Supplementary file 1 [file molecules-25-00009-s001.zip › 3.2.4_gcc-4.4.0.zip!/3.2.4_gcc-4.4.0/3.2.4_gcc-4.4.0/doc/octave/html/interpreter/Basic-Usage-and-Examples.html]

Basic Usage and Examples - Untitled


Next: Structure Arrays,
Up: Data Structures


---

#### 6.1.1 Basic Usage and Examples

Here are some examples of using data structures in Octave.

Elements of structures can be of any value type. For example, the three
expressions

```
     x.a = 1;
     x.b = [1, 2; 3, 4];
     x.c = "string";
```

create a structure with three elements. To print the value of the
structure, you can type its name, just as for any other variable:

```
     x
          => x =
             {
               a = 1
               b =
     
                 1  2
                 3  4
     
               c = string
             }
```

Note that Octave may print the elements in any order.

Structures may be copied just like any other variable:

```
     y = x
          => y =
             {
               a = 1
               b =
     
                 1  2
                 3  4
     
               c = string
             }
```

Since structures are themselves values, structure elements may reference
other structures. The following statements change the value of the
element `b` of the structure `x` to be a data structure
containing the single element `d`, which has a value of 3.

```
     x.b.d = 3;
     x.b
          => ans =
             {
               d = 3
             }
     
     x
          => x =
             {
               a = 1
               b =
               {
                 d = 3
               }
     
               c = string
             }
```

Note that when Octave prints the value of a structure that contains
other structures, only a few levels are displayed. For example,

```
     a.b.c.d.e = 1;
     a
          => a =
             {
               b =
               {
                 c =
                 {
                   1x1 struct array containing the fields:
     
                   d: 1x1 struct
                 }
               }
             }
```

This prevents long and confusing output from large deeply nested
structures. The number of levels to print for nested structures can be
set with the function `struct_levels_to_print`:

— Built-in Function: val = **struct\_levels\_to\_print** ()  
— Built-in Function: old\_val = **struct\_levels\_to\_print** (new\_val)  
> Query or set the internal variable that specifies the number of
> structure levels to display.

Functions can return structures. For example, the following function
separates the real and complex parts of a matrix and stores them in two
elements of the same structure variable.

```
     function y = f (x)
       y.re = real (x);
       y.im = imag (x);
     endfunction
```

When called with a complex-valued argument, `f` returns the data
structure containing the real and imaginary parts of the original
function argument.

```
     f (rand (2) + rand (2) * I)
          => ans =
             {
               im =
     
                 0.26475  0.14828
                 0.18436  0.83669
     
               re =
     
                 0.040239  0.242160
                 0.238081  0.402523
     
             }
```

Function return lists can include structure elements, and they may be
indexed like any other variable. For example,

```
     [ x.u, x.s(2:3,2:3), x.v ] = svd ([1, 2; 3, 4]);
     x
          => x =
             {
               u =
     
                 -0.40455  -0.91451
                 -0.91451   0.40455
     
               s =
     
                  0.00000   0.00000   0.00000
                  0.00000   5.46499   0.00000
                  0.00000   0.00000   0.36597
     
               v =
     
                 -0.57605   0.81742
                 -0.81742  -0.57605
     
             }
```

It is also possible to cycle through all the elements of a structure in
a loop, using a special form of the `for` statement
(see Looping Over Structure Elements).
